# CircGNG4 Promotes the Progression of Prostate Cancer by Sponging miR-223 to Enhance EYA3/c-myc Expression

**DOI:** 10.3389/fcell.2021.684125

**Published:** 2021-07-28

**Authors:** Shengxian Xu, Zhenpeng Lian, Siyang Zhang, Yong Xu, Hongtuan Zhang

**Affiliations:** Department of Urology, Tianjin Key Institute of Urology, The Second Hospital of Tianjin Medical University, Tianjin Medical University, Tianjin, China

**Keywords:** circGNG4, prostate cancer, miR-223, EYA3, c-myc, circular RNA

## Abstract

Patients diagnosed with prostate cancer often have a poor prognosis and limited treatment options, as the specific pathogenesis remains to be elucidated. Circular RNA (circRNA) is a type of non-coding RNA that interacts with microRNA (miRNA/miR) and transcription factors to regulate gene expression. However, little is known about specific circRNAs that serve roles in the pathogenesis of prostate cancer. Findings of the present study confirmed that circRNA G protein subunit γ 4 (circGNG4) was upregulated in prostate cancer tissues and cell lines. Knockdown of circGNG4 inhibited the malignant behavior of prostate cancer cells. Furthermore, bioinformatics were used to predict targeting interactions between circGNG4 or miR-223 and EYA transcriptional coactivator and phosphatase 3 (EYA3)/c-Myc mRNA. miR-223 inhibited the malignant behavior of prostate cancer cells, while EYA3/c-Myc had the opposite effect. circGNG4 enhanced the expression of EYA3/c-Myc by sponging miR-223 to promote the growth of prostate cancer tumors *in vivo*. In conclusion, the circGNG4/miR-223/EYA3/c-Myc regulatory pathway promoted the malignant progression of prostate cancer. The results of the present study may provide potential new targets for the diagnosis or treatment of prostate cancer.

## Introduction

Prostate cancer is one of the most prevalent forms of cancer affecting males, with a high degree of malignancy and poor prognosis (Logothetis et al., 2013). Prostate cancer is considered a malignant tumor requiring multidisciplinary treatment, as conventional therapy options often have limited success (Chang et al., 2014; Ge et al., 2020). Thus, further understanding of the specific pathogenesis of prostate cancer is required, which may lead to the development of novel therapeutic strategies.

Circular (circ)RNAs, microRNAs (miRNAs/miRs) and long non-coding (lnc)RNAs are all classed as non-coding RNAs (Wu et al., 2019). circRNAs are abnormally expressed in a variety of malignant tumors and are associated with the occurrence, proliferation, invasion and prognosis of tumors, including prostate cancer (Salzman, 2016; Chen et al., 2019; Hua et al., 2019; Vo et al., 2019). In prostate cancer, circ0005276 accelerates the progression of tumor cells by interacting with FUS RNA binding protein to transcriptionally activate X-linked inhibitor of apoptosis (Feng et al., 2019). p53-RNA binding motif protein 25-induced circRNA angiomotin like 1 promotes the development of prostate cancer via sponging miR-193a-5p (Yang et al., 2019). circRNA FOXO3 can promote the proliferation, migration and invasion of prostate cancer and increases chemoresistance to docetaxel (Shen et al., 2020).

The RNA-induced silencing complex (RISC) uses miRNAs as a template for the pairing of complementary mRNA bases of target genes to degrade the mRNA or inhibit its translation. circRNA can bind miRNAs, which in turn affects the specific binding to target genes, indirectly upregulating these target genes, thus affecting the occurrence and development of disease. Hence, circRNAs act as a sponge for the absorption of miRNA (Krol et al., 2010; Hayes et al., 2014; Jonas and Izaurralde, 2015; Rupaimoole and Slack, 2017). The circRNA/miRNA/mRNA axis has been demonstrated to be a regulator of multiple tumor-related pathways, with the ability to induce or inhibit tumorigenesis (Tay et al., 2014; Thomson and Dinger, 2016).

The eyes absent homolog 3 (EYA3) protein has demonstrated diverse roles in a range of biological processes, including cell development and tumorigenesis (Cancer, 2018). EYA3 is highly expressed in embryos and these expression levels decrease with maturation. The C-terminal of EYA3 is a highly conserved domain containing tyrosine, and the N-terminal domain is a poorly conserved domain containing silk/threonine phosphorylation sites (Wang et al., 2019). EYA3 is closely associated with tumor progression and low levels of treatment success (Vartuli et al., 2018), and interacts with cellular inhibitor of PP2A (PP2A) to increase the stability of c-Myc, thus promoting tumor development (Zhang et al., 2018). The level of EYA3 in breast cancers is elevated when compared with non-cancerous tissues, thereby promoting cell migration and invasion of breast cancer cells (Krueger et al., 2014).

In the present study, the existence and location of circRNA G protein subunit γ 4 (circGNG4) was identified in prostate cancer cells, and its upregulated expression was confirmed. Loss and gain of function studies determined that circGNG4 acted as an oncogene in prostate cancer. In addition, potential miRNA targets of circGNG4 were explored, and circGNG4 was found to inhibit miR-223. Inhibition of miR-223 led to the elevated expression of tumor-promoting factor EYA3 and c-Myc, thus enhancing the malignant behavior of prostate cancer. The findings of the present study extend the current understanding of the role and mechanism underlying circRNAs in prostate cancer progression.

## Results

### CircGNG4 Is Highly Expressed in Prostate Cancer

#### CircGNG4 Is Highly Expressed in Prostate Cancer

**FIGURE 1:**
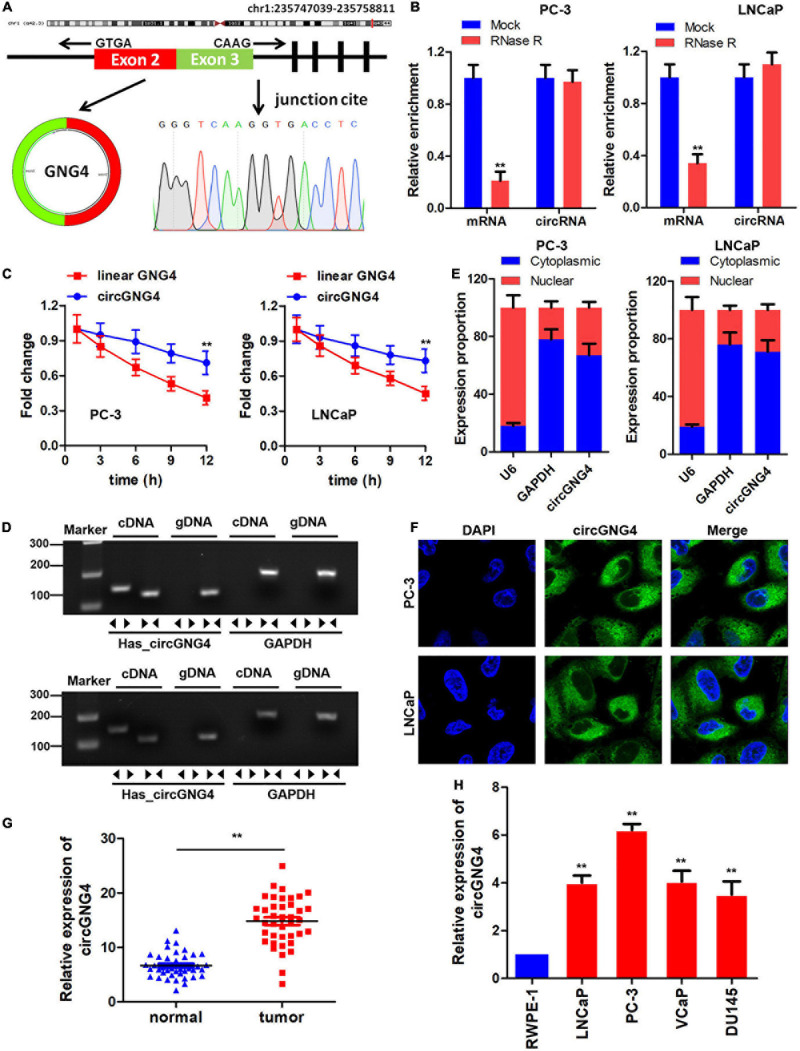
Study on circGNG4 cyclization mechanism. **(A)** The circRNA cyclization mechanism was spliced by exons 2 and 3, and the sequence was verified by sanger sequencing. **(B)** After RNase R treatment, linear and circRNA expression was detected to demonstrate stability. **(C)** After actinomycin treatment, the half-life of linear and circular GNG4 was detected. **(D)** Convergent and divergent primers were designed, and PCR was performed using cDNA and genomic DNA as the template for verification. **(E)** The nucleus and cytoplasm of cells was separated and quantitative PCR was used to detect the expression of circGNG4 in the nucleus and cytoplasm. **(F)** Fluorescence *in situ* hybridization was performed to detect the intracellular localization of circGNG4. **(H)** Expression of circGNG4 was detected in tumor tissues. **(G)** circGNG4 expression was detected in cell lines. **p < 0.01 vs. mock, linear GNG4, normal or RWPE-1 group. circRNA/circ, circular RNA; GNG4, G protein subunit γ 4.

An increasing number of studies have focused on the role of circRNAs in human cancers. In a previous study (Xia et al., 2018), microarray results demonstrated that hsa_circ_0017076 (circGNG4) was upregulated in prostate cancer; however, the existence and dysregulated expression of circGNG4 required further confirmation. In the present study, bioinformatics analysis was conducted and circGNG4 was found to be spliced by exon 2–3 of the GNG4 transcript. Sanger sequencing results confirmed this cyclization mechanism (Figure 1A). Subsequently, the expression of linear and circRNA was determined following RNase R treatment to establish the stability of circGNG4. Experimental results demonstrated that the circular form of GNG4 was stable in both PC-3 and LNCaP cells (Figure 1B). In addition, following treatment with actinomycin, the half-life of linear and circular circGNG4 was investigated, and circGNG4 had a longer half-life and high stability compared with that of linear GNG4 (Figure 1C). Thereafter, convergent and divergent primers were designed, and PCR was performed using cDNA and genomic (g)DNA as the respective templates. The findings of the present study demonstrated that circGNG4 could be found in cDNA but not gDNA (Figure 1D). Furthermore, the location of circRNAs may highlight their involvement in regulatory mechanisms of other circRNAs. Quantitative (q)PCR and fluorescence *in situ* hybridization (FISH) experiments were used to identify the location of circGNG4, and the results demonstrated that circGNG4 was mostly located in the cytoplasm (Figures 1E,F). The expression of circGNG4 in prostate cancer was detected, and results demonstrated that circGNG4 was significantly increased in prostate cancer when compared with the control group (Figure 1G). Further verification was performed at the cellular level, using normal prostatic epithelial cells RWPE-1 and prostate cancer cells. circGNG4 was highly expressed in prostate cancer cells (Figure 1H).

### CircGNG4 Promotes Prostate Cancer Progression *in vitro*

**FIGURE 2:**
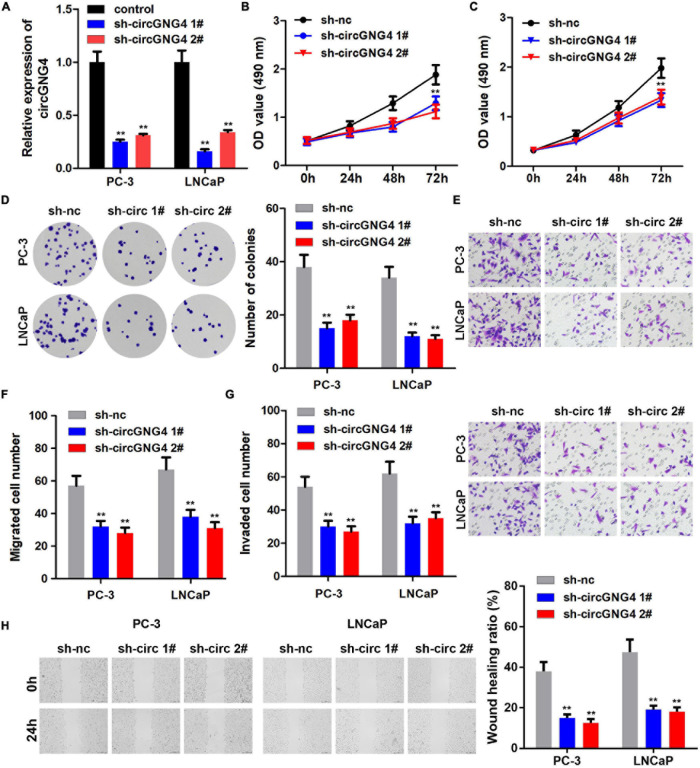
CircGNG4 promoted the progression of prostate cancer *in vitro*. **(A)** circGNG4 expression level was detected and the results showed that plasmid knockout could significantly reduce circGNG4 expression. **(B,C)** Detection of cell proliferation after circGNG4 knockout was determined using a Cell Counting Kit-8 assay. **(D)** Experimental detection of cell clone formation after circGNG4 knockout. **(E–G)** Cell migration and invasion were investigated using Transwell assays. **(H)** Cell migration was investigated using a wound healing assay. **p < 0.01 vs. sh-NC group. circRNA/circ, circular RNA; GNG4, G protein subunit γ 4; sh, short hairpin RNA; NC, negative control.

Following the confirmation of the upregulation of circGNG4 in prostate cancer, the biological functions of circGNG4 in the development of prostate cancer were explored using cell proliferation, cell migration and clone formation experiments. The detection results of circGNG4 expression demonstrated that the short hairpin RNA (shRNA/sh) plasmid effectively reduced the expression of circGNG4 (Figure 2A). Cell Counting Kit-8 (CCK-8) assay results demonstrated that circGNG4 knockdown inhibited prostate cancer cell proliferation (Figures 2B,C). In addition, knockdown of circGNG4 inhibited the clonal formation ability of prostate cancer cells (Figure 2D). Transwell and wound healing results showed that circGNG4 knockdown could inhibit the invasion and migration of prostate cells (Figures 2E–H).

### CircGNG4 Spongesmir-223 in Prostate Cancer Cell

**FIGURE 3:**
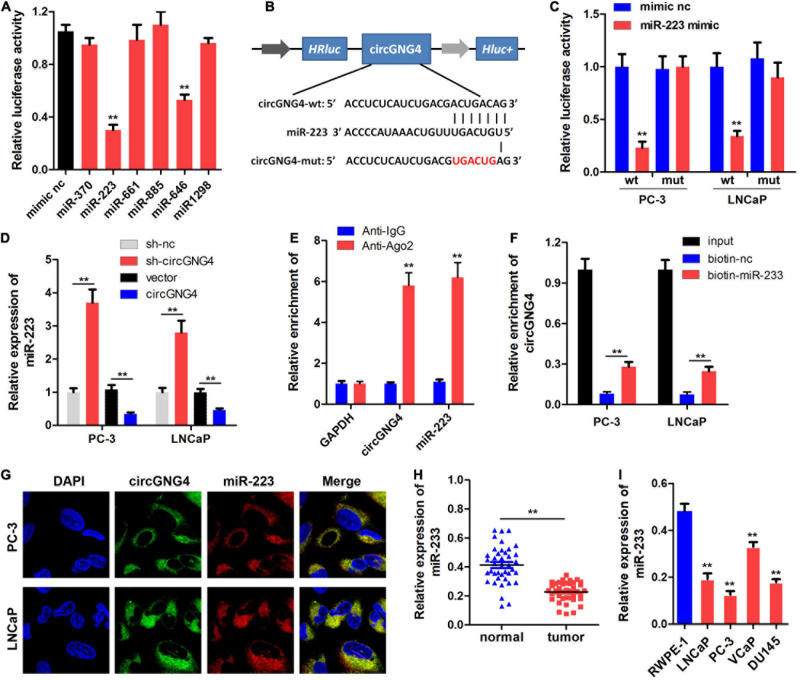
CircGNG4 spongesmiR-223in prostate cancer cells. **(A)** Candidate miRNAs bound to circGNG4 were screened using a dual luciferase reporter assay. **(B)** The binding site between circGNG4 and miR-223 was predicted using bioinformatics analysis. **(C)** Dual luciferase reporter assay confirmed the binding between circGNG4 and miR-223. **(D)** After circGNG4 knockdown or overexpression, miR-223 expression level was detected by quantitative PCR. **(E)** RNA immunoprecipitation assay using Ago-2 antibody was performed to detect the interaction between circGNG4 and miR-223 with Ago-2 protein. **(F)** RNA pull-down using a biotin-labeled miR-223 probe was carried out to detect the interaction between circGNG4 and miR-223. **(G)** Fluorescence *in situ* hybridization was performed to detect the co-localization between circGNG4 and miR-223. **(H)** Expression of miR-223 in prostate cancer tissues was detected. **(I)** Expression of miR-223 in prostate cancer cell lines was detected. **p < 0.01 vs. mimic NC, anti-IgG and RWPE-1 group. circRNA/circ, circular RNA; GNG4, G protein subunit γ 4; sh, short hairpin RNA; NC, negative control; miR/miRNA, microRNA.

The specific molecular mechanism underlying circRNAs remains to be fully elucidated. It has previously been established that circRNAs can sponge miRNAs to block their regulation of target genes (Shang et al., 2019). In the present study, the interactions of potential miRNAs with circGNG4 were predicted using bioinformatics analysis, and miR-223 and miR-646 were selected for further experimental analysis. The luciferase assay results indicated that miR-223 and miR-646 overexpression inhibited the luciferase activity of vectors carrying the sequence of circGNG4 (Figure 3A). As miR-223 has been reported to have an anti-cancer effect in prostate cancer (Kurozumi et al., 2016), it was selected for further research. The predicted binding site of miR-233 to circGNG4 was demonstrated in Figure 3B. A luciferase reporter assay was performed using the reporter plasmid cloned with either the wild-type (WT) or mutant type (mut) binding sequence. miR-223 notably inhibited the luciferase activity of the reporter vector carrying the WT version of the binding sequence, but this was not observed in the mut version. This result indicated the positive interaction between circGNG4 and miR-223 (Figure 3C). It was also demonstrated that overexpression of circGNG4 inhibited the expression of miR-223, while inhibition of circGNG4 promoted miR-223 expression (Figure 3D). Ago2 is the main component of the RISC, and the RNA immunoprecipitation (RIP) assay, using an antibody to target Ago2, confirmed that circGNG4 and miR-223 both interacted with Ago2 (Figure 3E). RNA pull-down using a biotin-labeled miR-223 probe demonstrated that miR-223 could bind to circGNG4 (Figure 3F). FISH analysis was carried out to detect the location of circGNG4 and miR-223, and these results indicated an interaction between circGNG4 and miR-223 in the cell cytoplasm (Figure 3G). Furthermore, miR-223 expression was also lower in prostate cancer tissues and cell lines (Figures 3H,I).

### miR-223 Targets EYA3 in Prostate Cancer Cell

**FIGURE 4:**
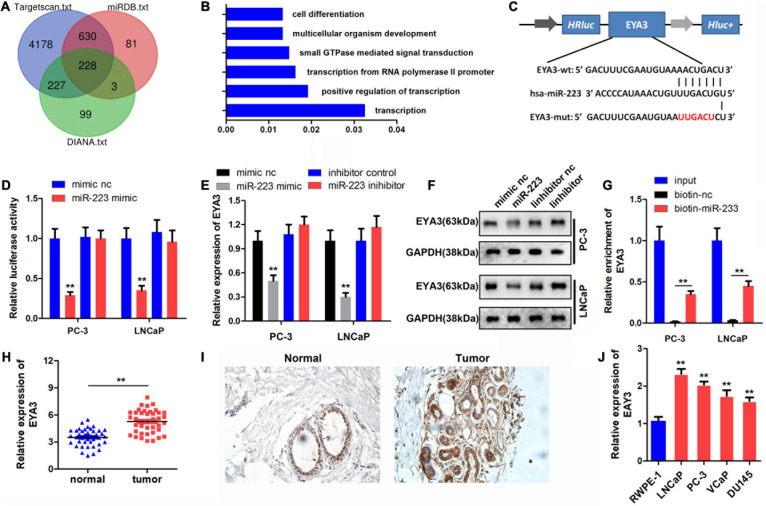
Targeted binding of miR-223 to EYA3. **(A)** TargetScan, miRDB and DIANA databases were used to predict the target of miR-223 and the target genes were intersected. **(B)** Functional enrichment analysis of target genes. **(C)** The binding sites and mutations of miR-223 and EYA3 are shown. **(D)** Dual luciferase reporter assay confirmed the binding between miR-223 and EYA3. **(E,F)** Western blot was performed to detect the expression of EYA3 after the overexpression or knockdown of miR-223. **(G)** RNA pull-down assay verified the binding between miR-223and EYA3. **(H,I)** Analysis of EYA3 expression in prostate cancer tissues using qPCR and immunohistochemistry. **(J)** Expression of EYA3 in prostate cancer lines was detected by qPCR. **p < 0.01 vs. mimic NC and RWPE-1 group. miR, microRNA; EYA3, eyes absent homolog 3; qPCR, quantitative PCR; NC, negative control.

To investigate the downstream target genes of miR-223, the binding target genes were determined using TargetScan 7.2, miRDB and DIANA bioinformatics tools. A total of 228 genes were obtained following Venn analysis (Figure 4A). Gene Ontology (GO) enrichment was performed by Metascape software^1^ (Figure 4B), and EYA3 was selected for further study. The binding site between miR-223 and EYA3 was revealed (Figure 4C). Results of the luciferase assay indicated that miR-223 binds directly to EYA3 (Figure 4D). Western blot analysis indicated that miR-223 inhibited the expression of EYA3, while miR-223 inhibition could promote that (Figures 4E,F). The RNA pull-down results revealed that biotin-labeled miR-223 could directly target EYA3 (Figure 4G). Furthermore, EYA3 was highly expressed in prostate cancer tissues, which was demonstrated by qPCR and immunohistochemistry (IHC) detection (Figures 4H,I), and EYA3 was also highly expressed in prostate cancer cell lines (Figure 4J).

### Overexpression of miR-223 and Knockdown of EYA3 Both Could Reverse the Oncogenic Effect of circGNG4

**FIGURE 5:**
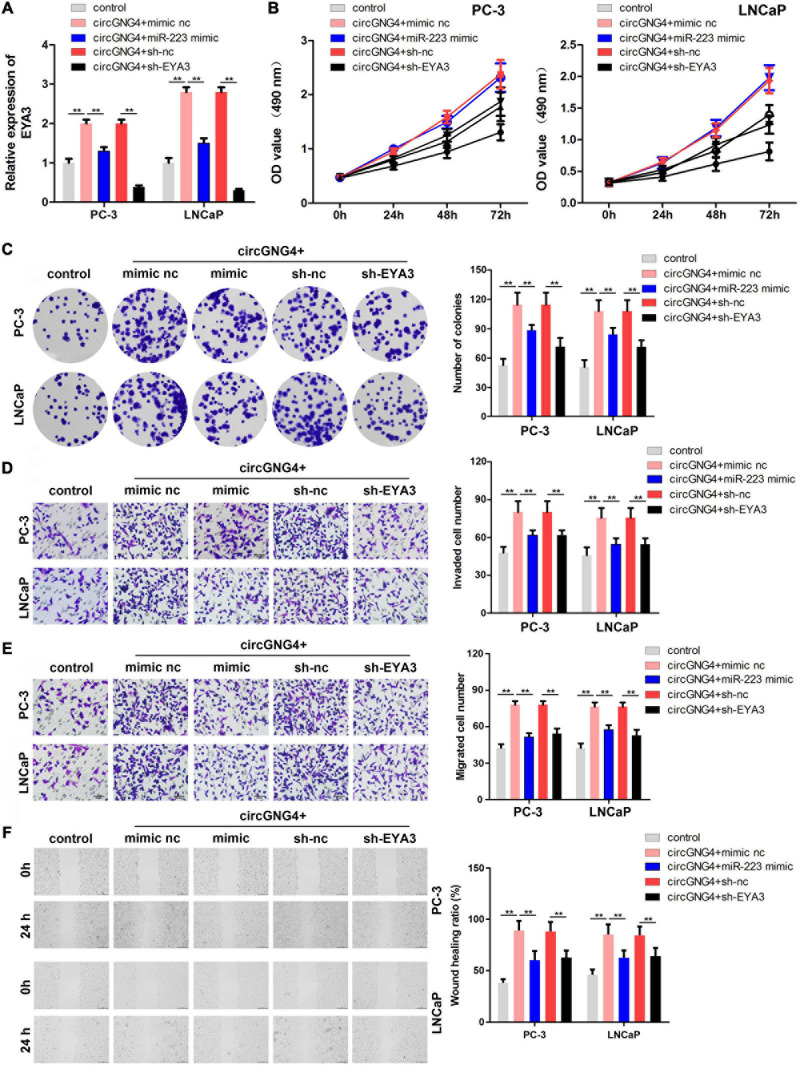
Overexpression of miR-223 and knockdown of EYA3 reversed the oncogenic effect of circGNG4. **(A)** EYA3 expression levels were detected by quantitative PCR analysis **(B)** Detection of cell proliferation after circGNG4 knockout was determined using a Cell Counting Kit-8 assay. **(C)**. Experimental detection of cell clone formation after circGNG4 knockout. **(D,E)**. Cell migration and invasion were investigated using Transwell assays. **(F)** Cell migration was investigated using a wound healing assay. **p < 0.01. circRNA/circ, circular RNA; GNG4, G protein subunit γ 4; miR, microRNA.

Mechanistic studies have indicated the binding between miR-223 and circGNG4, and the binding between miR-223 and EYA3. To further confirm these interactions, rescue experiments were performed. The prostate cancer cells were transfected with circGNG4 overexpressing vector, miR-223 mimics or sh-EYA3 vectors. qPCR analysis initially confirmed that circGNG4 increased the expression level of EYA3. miR-223 mimic reversed this elevation and sh-EYA3 significantly decreased the expression level of EYA3 (Figure 5A). Malignant behavior analysis of prostate cancer cells was further evaluated by CCK-8, colony formation, transwell and wound healing assay. The results indicated that miR-223 overexpression or EYA3 knockdown both can reverse the function of circGNG4 on the prostate cancer cell proliferation, migration and invasion (Figures 5B–F).

### CircGNG4 Promotes the Growth of Prostate Cancer *in vivo*

**FIGURE 6:**
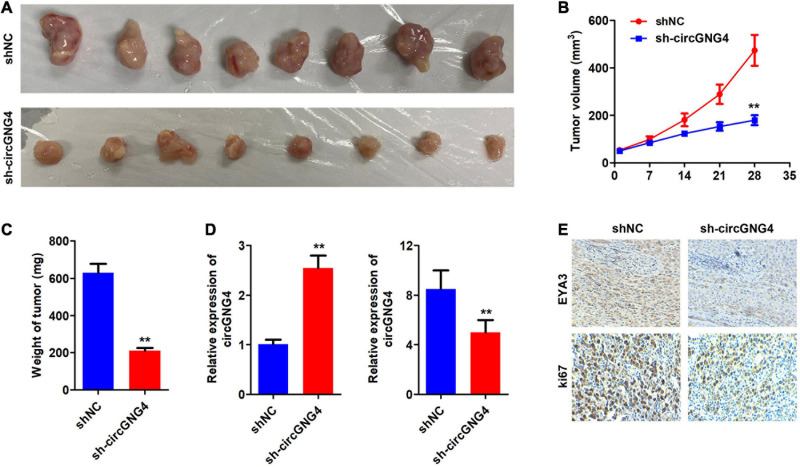
CircGNG4 promotes tumor growth *in vivo*. **(A)** Gene Expression Profiling Interactive Analysis (http://gepia.cancer-pku.cn/) database was used to determine genes associated with EYA3. **(B)** Western blot was used to detect the expression of c-Myc after EYA3 knockdown or overexpression. **(C)** The half-life of c-Myc mRNA was detected by quantitative PCR. **(D,E)** Western blot detected the expression of EYA3, c-Myc and cyclin proteins. **p < 0.01 vs. shNC group.

We further investigated the carcinogenic effects of circGNG4 at the animal level. The results of animal experiments showed that knockdown of circGNG4 could inhibit tumor growth and decrease the tumor weight (Figures 6A–C). We detected the expression level of circGNG4 and miR-223 to verify if we have successfully knockdown circGNG4. The qPCR analysis determined that the expression of circGNG4 in the sh-circGNG4 group was notably lower than that in the shNC group while miR-223 level in sh-circGNG4 group was higher than that in shNC group (Figure 6D). IHC was further performed and indicated that circGNG4 knockdown inhibited the ki67 and EYA3 expression in the tumor tissues (Figure 6E).

### The CircGNG4/miR-223/EYA3 Axis Regulates the Prostate Cancer Cell Cycle by Regulating c-Myc

**FIGURE 7:**
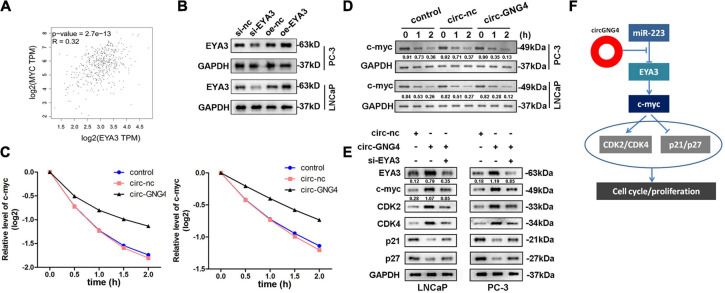
CircGNG4/miR-223/EYA3/c-myc axis mechanism. **(A)** GEPIA (http://gepia.cancer-pku.cn/) database was used to find the related gene of EYA3. **(B)** Western blot was used to detect the expression of c-myc after EYA3 knockdown or overexpression. **(C)** The half-life of c-myc mRNA was detected by qPCR. **(D,E)** Western blot detected the expression of EYA3, c-myc and cyclin protein expressions. **(F)** The diagram was established as showed.

A previous study reported that EYA3 interacts with PP2A to regulate the stability of c-Myc mRNA in breast cancer. However, the interaction between EYA3 and c-Myc in prostate cancer remains to be elucidated. The Gene Expression Profiling Interactive Analysis database^2^ was searched in order to distinguish a correlation between EYA3 and c-Myc. Interestingly, EYA3 was positively correlated with c-Myc (Figure 7A). Western blot analysis was used to confirm this interaction. It was found that EYA3 silencing inhibited c-Myc expression, while overexpression of EYA3 promoted c-Myc expression (Figure 7B). As EYA3 was revealed to regulate the stability of c-Myc, it was hypothesized that circGNG4 may also affect the stability of c-Myc. As demonstrated in Figure 7C, circGNG4 overexpression promoted the stability of c-Myc mRNA. Moreover, western blot analysis further confirmed that circGNG4 promoted the protein expression of c-Myc (Figure 7D). Further detection of cell cycle-related proteins revealed that the overexpression of circGNG4 upregulated the expression of cyclin CDK2/CDK4 and inhibited the expression of p21 and p27, while knockdown of EYA3 reversed the promotion of circGNG4 (Figure 7E). The findings indicated in Figure 7F demonstrated novel functions of circRNA and builds upon the potential role of the circGNG4/miR-223/EYA3/c-Myc signaling pathway in prostate cancer.

## Discussion

Results of the present study confirmed the existence and elevated expression of a novel circRNA (circGNG4) in prostate cancer. Both loss and gain of function studies determined the oncogenic role of circGNG4 in prostate cancer. In previous studies, a number of circRNAs have been found to play critical roles in prostate cancer progression. For instance, circRNA nucleolar and coiled-body phosphoprotein 1, circRNA formin 1, circ-0016068, circ_0088233 and circ0005276 are all upregulated in prostate cancer and promote the development of prostate cancer (Feng et al., 2019; Chen et al., 2020; Deng et al., 2020; Li et al., 2020; Shan et al., 2020). The findings of the present study extend the current understanding of the function of circRNAs in prostate cancer.

A number of circRNA molecules contain miRNA response elements and act as competitive endogenous RNAs to sponge and inhibit the function of miRNA (Sardina et al., 2017). miRNAs are involved in regulating the signaling pathways associated with tumor growth. Studies have found that the abnormal expression of multiple miRNAs in prostate cancer tissues is closely associated with the occurrence and development of prostate cancer (Fabris et al., 2016). miR-409-3p/-5p promotes the growth and metastasis of human prostate cancer (Josson et al., 2014). miR-141 attenuates the proliferation of prostate cancer stem cells and metastasis by binding with several metastasis related genes (Liu et al., 2017). miR-424 affects the ubiquitination and activation of STAT3 to promote prostate cancer development (Dallavalle et al., 2016). In the present study, miR-233 was predicted to bind with circGNG4, and miR-223 has been determined to inhibit prostate cancer progression by targeting ETS transcription factor ERG (ERG) and integrin subunit α 3 (ITGA3)/integrin subunit β 1 (ITGB1) signaling (Kurozumi et al., 2016; Wei et al., 2020). It is therefore hypothesized that circGNG4 exerts its oncogenic role via sponging miR-223, which blocks the anti-cancer effect of miR-233. Further experimental research confirmed this hypothesis. However, in addition to ERG and ITGA3/ITGB1 signaling, alternative target genes of miR-233 remain to be elucidated in prostate cancer. In the present study, bioinformatics analysis was carried out using three online tools, including TargetScan 7.2, miRDB and DIANA followed by GO enrichment analysis. EYA3 was selected, which has recently been found to regulate the stability of oncogene c-Myc (Zhang et al., 2018).

EYA3 demonstrated dynamic changes in higher biological development and tumorigenesis (Miller et al., 2010). The C-terminal of EYA3 is highly conserved, while the N-terminal is poorly conserved, which is closely related to tumor progression and treatment tolerance. EYA3 promotes chemotherapy resistance of osteosarcoma by regulating miR-708. EWS RNA binding protein 1/Fli-1 proto-oncogene, ETS transcription factor regulates EYA3 in Ewing sarcoma via modulation of miR-708, resulting in increased cell survival and chemoresistance.

As previously mentioned, EYA3 can modulate the stability of c-Myc in breast cancer. In the present study, EYA3 was confirmed to promote the stability of c-Myc in prostate cancer cells (Vartuli et al., 2018). c-Myc is well established as a key oncogene, which encodes proteins that are phosphorylated in the nucleus (Dang, 2012). c-Myc plays an important role in maintaining normal cell function, and is involved in cell proliferation, differentiation and apoptosis, including in prostate cancer cells (Ellwood-Yen et al., 2003). c-Myc is closely associated with human tumors, and the abnormal expression of the proto-oncogene c-Myc and its corresponding protein products can be detected in many tumor tissues. Moreover, c-Myc can mediate the effect of prostate transmembrane protein, androgen induced 1 and inhibit the expression of p21 (Liu et al., 2011). c-Myc inhibits FOXO3a-mediated activation of the p27 promoter in multiple cell lines (Chandramohan et al., 2008). In the present study, the circGNG4/miR-223/EYA3 axis was demonstrated to regulate the expression of CDK2/CDK4 and p21/p27 through c-Myc, which is consistent with the findings of previous studies.

The present study has certain limitations. Although circGNG4 modulates the migration or invasion of prostate cancer cells, this has not been verified in animal models, and a precise mechanism underlying the effect of circGNG4 remains to be established. Regulation of the epithelial-mesenchymal transition process and critical metastasis-related genes will be studied in following experiments.

In conclusion, circGNG4 was revealed to be upregulated and played an oncogenic role in prostate cancer. Mechanistically, circGNG4 sponged miR-223 to promote EYA3 expression, which led to the modulation of c-Myc. The circGNG4/miR-223/EYA3/c-Myc signaling axis may therefore be crucial for the development of prostate cancer, and may act as a potential therapeutic target for prostate cancer.

## Materials and Methods

### Specimen Collection

Cancer and adjacent normal tissues were collected from 40 prostate cancer patients admitted to The Second Hospital of Tianjin Medical University. The tissues were stored in liquid nitrogen immediately after resection. The study was approved by the Ethics Committee of The Second Hospital of Tianjin Medical University. Written informed consent was obtained from each patient.

### Cell Culture

Prostate cancer cell lines, PC-3, LNCaP, VCaP, and DUL145, and the human normal prostatic epithelial cell line, RWPE-1, were purchased from the American Type Culture Collection. Cells were recovered from liquid nitrogen and maintained in DMEM (Invitrogen; Thermo Fisher Scientific, Inc., United States) containing 10% FBS supplemented with 100 units/ml penicillin and 100 g/ml streptomycin (Beyotime Institute of Biotechnology, Shanghai, China), and incubated at 37°C with 5% CO_2_.

### Cell Transfection

miR-223 mimic, mimic negative control (NC), miR-223 inhibitor and inhibitor NC were purchased from Shanghai GenePharma Co., Ltd. (Shanghai, China). The pSilencer vector carrying sh-circGNG4 and sh-EYA3, and the pcD-ciR vector overexpressing circGNG4 were established. Cells were transfected with the corresponding plasmids using a Lipofectamine^®^ 3000 kit (Invitrogen; Thermo Fisher Scientific, Inc.) according to the manufacturer’s instructions. After 24 h of culture, cell culture medium was replaced with DMEM containing 10% FBS.

### Quantitative Polymerase Chain Reaction (qPCR)

TRIzol^®^ (Invitrogen; Thermo Fisher Scientific, Inc., United States) was used for RNA extraction, according to the manufacturer’s protocol. The RNA was reverse-transcribed into cDNA using a reverse transcription kit (Takara Bio, Inc., Japan). The expression of genes was detected with a fluorescence quantitative PCR kit (Nanjing Jiancheng Bioengineering Institute, China) on a FAST7500 flow cytometry system (BD Biosciences, United States). U6 and GAPDH were used as internal controls for miRNAs and mRNAs, respectively. The experiment was repeated at least three times. The primers used are as follows: RT primer for miR-223: 5′GTCGTATCCAG TGCAGGGTCCGAGGTGCACTGGATACGACACGUACU 3′; miR-223 forward: 5′TGCGGUGUCUUGCAGGCCGUCAG3′; miR-223 reverse: 5′CCAGTGAGGGTCCGAGGT3′; U6 forward: 5′CTCG CTT CGGCAG CACA3′; U6 reverse: 5′AAC GCTTCACGAATTTGCGT3′; circGNG4 forward: 5′AACATG CTGCTGGATAAATCTGG3′; circGNG4 reverse: 5′TGTATCA CATCGTACCATGCCT3′; EYA3 forward: 5′AACATGCTGCT GGATAAATCTGG3′; EYA3 reverse: 5′TGTATCACATCGTA CCATGCCT3′; GAPDH forward: 5′TGGCAAGACAACGTG AAAGA3′; GAPDH reverse: 5′AACTGGGAAAATGCATCT GG3′.

### Western Blot

Proteins were extracted using RIPA buffer (Beyotime Institute of Biotechnology, Shanghai, China) supplemented with a protease inhibitor. The BCA method (Pierce; Thermo Fisher Scientific, Inc., Rockford, United States) was used to detect the amount of protein. Proteins were separated by gel electrophoresis (SDS-PAGE) and transferred to PVDF membranes (MilliporeSigma, United States). Following blocking in 5% skimmed milk powder, the PVDF membrane was incubated with the following primary antibodies: EYA3 (Abcam, United States), c-Myc (ProteinTech Group, Inc., China), CDK2 (ProteinTech Group, Inc., China), CDK4 (ProteinTech Group, Inc., China), p21 (Abcam, United States), p27 (Abcam, United States) and GAPDH (ProteinTech Group, Inc., China) at 4°C overnight. The relative expression levels of specific proteins in cells were detected with a horseradish peroxidase-labeled secondary antibody, which was incubated with the membrane at room temperature for 2 h. An enhanced chemiluminescence kit was used for imaging according to the manufacturer’s instructions.

### CCK-8 Assay

Following transfection, the logarithmic cells were collected and seeded into a 96-well plate and cultured at 37°C in 5% CO_2_ for 72 h. The CCK-8 assay was performed at 0, 24, 48, and 72 h. At each time point, the medium was replaced and 10 μl CCK-8 was added to each well. Following incubation at 37°C for 4 h, the optical density was measured at 490 nm. The experiment was repeated at least three times.

### Clone Formation Assay

Following transfection, cells of each experimental group were digested with trypsin while in the logarithmic growth phase, and the cell suspension was prepared. Subsequently, cells were seeded into six-well plates at 500–1,000 cells/well and cultured for 2 weeks in an incubator at 37°C with 5% CO_2_. After 2 weeks of incubation, cells were fixed with 5 ml of 4% paraformaldehyde for 15 min, and stained with GIMSA for 20 min. The colonies were counted.

### Transwell Assay

Following transfection, cells were washed twice with PBS and resuspended in serum-free medium. A total of 100–200 μl of cell suspension was added to the upper chamber of the Transwell insert containing Matrigel for invasion analysis. Cell suspension was added to the upper chamber without Matrigel for migration analysis. A total of 600 μl complete medium supplemented with 10% serum was added to the Transwell chamber. Following 24 h of culture, cells in the upper chamber were removed with cotton swabs, fixed with formaldehyde, and stained with crystal violet (Sigma-Aldrich; Merck KGaA). Cells were photographed using a microscope (Olympus Corporation, Japan).

### Dual Luciferase Assay

The dual luciferase reporter assay was performed using the psiCHECK reporter system (Thermo Fisher Scientific, Inc., United States). WT or mut sequences of circGNG4 and EYA3 were cloned into a psiCHECK2 plasmid. Prostate cancer cells (2 × 10^4^ cells/well) were cultured overnight in 24-well plates. Cells were transfected with the WT or mut reporter vector, along with the miR-223 mimic or mimic NC using Lipofectamine^®^ 3000 (Invitrogen; Thermo Fisher Scientific, Inc., United States). PRL-TK (TK-driven *Renilla* luciferase expression vector) was co-transfected as an internal control. Cells were harvested and the luciferase activity was detected using a Dual-Luciferase Detection kit (Promega Corporation, WI, United States) following 48 h of transfection.

### RNA-Fluorescence *in situ* Hybridization

Hybridization of circGNG4 was carried out using a Cy2-labeled probe, and the miR-223 was carried out using a Cy5-labeled probe according to the manufacturer’s protocol (Shanghai GenePharma Co., Ltd., Shanghai, China). DAPI was added to stain the cell nucleus. The subcellular distribution of circGNG4 and miR-223 in prostate cancer cells was observed using a confocal laser scanning microscope (FV1000; Olympus Corporation, Japan).

### RNA Pull Down

Biotin-labeled miR-223 and corresponding NC probes were obtained from Shanghai GenePharma Co., Ltd. (Shanghai, China). The biotin-labeled miR-223 probe was incubated with streptavidin-coated beads at room temperature for 3 h to obtain the probe-coated beads. PC-3 and LCNaP cells were lysed, and the lysate was incubated with biotin-miR-223 probe at 4°C for 12 h. Following elution from the beads, the complex was purified by TRIzol and the amount of circGNG4 and EYA3 was evaluated using qPCR.

### Immunohistochemical (IHC)

Paraffin-embedded sections were rinsed in distilled water and soaked in PBS for 5 min for dewaxing. The sections were incubated with 3% H_2_O_2_ at room temperature for 10 min to eliminate endogenous peroxidase activity, and subsequently washed with PBS three times. Thereafter, sections were blocked in 5% normal goat serum and incubated at room temperature for 10 min. Sections were incubated with the following primary antibodies: Ki67 (1:800; ProteinTech Group, Inc., China) and EYA3 (1:500; Abcam, United States) overnight at 4°C. Following the primary incubation, HRP-conjugated secondary antibodies were added and sections were incubated for 1 h at room temperature. A DAB kit (Beyotime Institute of Biotechnology, Shanghai, China) was used to visualize the positive staining, and slides were observed and imaged under a microscope (Olympus Corporation, Japan).

### Xenograft Model

The PC-3 cells in the logarithmic growth phase were digested, centrifuged, dumped out of the old medium, and resuspended in PBS. Then the cell concentration was adjusted to 1 × 10^6^/mL, and 0.1 mL was injected subcutaneously in BALB/c nude mice (*n* = 6). The blank group was inoculated with 0.1 mL PBS. After planting, the tumor size was measured once every week after the obvious tumor nodules appeared at the inoculation site. The tumor size was measured with a vernier caliper, the longest diameter of the tumor (a) and the largest vertical transverse diameter (b) were measured, and the tumor volume was calculated according to the formula V (mm^3^) = ab^2^/2. At the end of the experiments, the mice were euthanized with intraperitoneal injection of 3% pentobarbital sodium (160 mg/kg). The tumors were removed, pictured and weighted.

### Statistical Analysis

The results were repeated at least three times. In terms of mean ± standard deviation. The Student’s *t*-test was used for the inter-group comparison, and one-way ANOVA was used to evaluate its significance. *P* < 0.05 was considered statistically significant.

## Data Availability Statement

The raw data supporting the conclusions of this article will be made available by the authors, without undue reservation.

## Ethics Statement

The studies involving human participants were reviewed and approved by Ethics Committee of The second Hospital of Tianjin Medical University. The patients/participants provided their written informed consent to participate in this study. The animal study was reviewed and approved by Ethics Committee of The second Hospital of Tianjin Medical University.

## Author Contributions

SX and ZL conducted the experiments. SZ helped analyze the data. YX and HZ designed the experiments and wrote the manuscript. All authors contributed to the article and approved the submitted version.

## Conflict of Interest

The authors declare that the research was conducted in the absence of any commercial or financial relationships that could be construed as a potential conflict of interest.

## Publisher’s Note

All claims expressed in this article are solely those of the authors and do not necessarily represent those of their affiliated organizations, or those of the publisher, the editors and the reviewers. Any product that may be evaluated in this article, or claim that may be made by its manufacturer, is not guaranteed or endorsed by the publisher.

## Footnotes

1
http://metascape.org/

2
http://gepia.cancer-pku.cn/index.html
